# Acute Conjunctivitis among Patients Visiting the Outpatient Department of Ophthalmology in a Tertiary Care Centre

**DOI:** 10.31729/jnma.8391

**Published:** 2024-01-31

**Authors:** Ram Shrestha, Roshija Khanal Rijal, Neyaz Kausar, Oshan Shrestha, Sagar Rajkarnikar, Raina Chaudhary, Kabindra Lal Shrestha, Sitaram Khadka

**Affiliations:** 1 Department of Ophthalmology, Shree Birendra Hospital, Chhauni, Kathmandu, Nepal; 2 Department of Ophthalmology, Shreekrishna Netralaya, Rudrapath, Bhairahawa, Nepal; 3 Department of Ophthalmology, Nepal Eye Hospital, Tripureshwor, Kathmandu, Nepal; 4 Nepalese Army Institute of Health Sciences, Sanobnaryang, Kathmandu, Nepal; 5 Department of Microbiology, Nepalese Army Institute of Health Sciences, Sanobharyang, Kathmandu, Nepal; 6 Health Service Division, Shree Birendra Hospital, Chhauni, Kathmandu, Nepal

**Keywords:** *conjunctivitis*, *disease outbreaks*, *enterovirus*

## Abstract

**Introduction:**

Conjunctivitis is a highly prevalent ocular disease that flares up every year. The humidity and high temperature favour the causative agents responsible for the epidemic. Acute infective conjunctivitis may be either viral or bacterial, a distinct type of condition with unique clinical features and treatment approaches. The aim of the study was to find out the prevalence of acute conjunctivitis among patients visiting the outpatient Department of Ophthalmology in a tertiary care centre.

**Methods:**

This descriptive cross-sectional study was conducted among the patients visiting the outpatient Department of Ophthalmology. Data of 30 August 2023 to 30 September 2023 was collected between 21 November 2023 to 24 November 2023. All patients presenting in the Ophthalmology Department having complete hospital record were included in the study. Patients having missing data on the medical records of the hospital were excluded. A convenience sampling method was used. The point estimate was calculated at a 95% Confidence Interval.

**Results:**

Among 5,507 patients, acute conjunctivitis was seen in 1240 (22.52%) (21.42-23.62, 95% Confidence Interval). The majority were male 732 (59.03%) and adults 760 (61.29%) with a mean age of 32.56±18.74 years.

**Conclusions:**

The prevalence of conjunctivitis among patients visiting the outpatient Department of Opthalmology was found to be higher than other studies done in similar settings.

## INTRODUCTION

Conjunctivitis is the most prevalent ocular disease occurring globally.^1^ Acute infective conjunctivitis may be either viral or bacterial. According to the onset and the clinical response, viral conjunctivitis may be categorised into acute, hyperacute, and chronic types.^2^

While most bacterial conjunctivitis are self-limiting types with mucopurulent discharge, some cases have the potential for ocular morbidity.^3^ Although self- limiting, antimicrobial therapy has shown clinical effectiveness, which has reduced distress, symptoms, duration, and contagious spread.^4,5^ Adenovirus affects the conjunctiva and cornea as well, while enterovirus has more effects on the conjunctiva.

The aim of the study was to find out the prevalence of acute conjunctivitis among patients visiting the outpatient Department of Ophthalmology in a tertiary care centre.

## METHODS

A descriptive cross-sectional study was conducted in the outpatient Department of Ophthalmology at Shree Birendra Hospital (SBH). Ethical approval was received from the Institutional Review Committee (Reference number: 245). Data of 30 August 2023 to 30 September 2023 was collected between 21 November 2023 to 24 November 2023. All patients presenting in the Ophthalmology Department having complete hospital record were included in the study. Patients with missing data on the medical records of the hospital were excluded. A convenience sampling method was used. The sample size was calculated using the following formula:


$$\begin{array}{ll} \text{n} & ={\text{Z}}^{2}\times \frac{\text{p}\times \text{q}}{{\text{e}}^{\text{2}}}\\ & =1.96^{2}\times \frac{0.50\times 0.50}{0.02^{2}}\\ & =2,401 \end{array}$$

Where,

n = minimum required sample size
Z = 1.96 at 95% Confidence Interval (CI)
p = prevalence taken as 50% for maximum sample size calculation
q = 1-p
e = margin of error, 2%

The calculated sample size was 2,401. As the convenience sampling method was used, the sample was doubled and became 4,802. However, 5,507 patients were included in the study.

Demographic details, clinical signs and symptoms of the patients, and co-morbidities were noted.

Data were entered and analyzed using IBM SPSS Statistics version 21.0. The point estimate was calculated at a 95% CI.

## RESULTS

**Figure 1:**
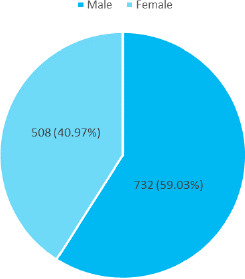
Gender-wise distribution (n= 58).

Out of 5507 patients, 1,240 (22.52%) (21.42-23.62, 95% CI) presented with acute conjunctivitis. It was more predominant in male 732 (59.03%) (Figure 1).

The mean age of patient was 32.56±18.74 years. The majority of the patients were residents of Kathmandu district 910 (73.39%), followed by Bhaktapur and Lalitpur districts (Table 1).

**Table 1:** Demographic evaluation of the patients (n= 1240).

Variables		n (%)
Age group (years)	0-17	380 (30.65)
	18-59	760 (61.29)
	≥60	100 (8.06)
Location	Kathmandu	910 (73.39)
	Bhaktapur	217 (17.50)
	Lalitpur	113 (9.11)

The most frequent presenting complaints after red eye were redness, eye swelling, watering, discharge, and discomfort. On clinical evaluation, congestion was present among all the cases whereas serous exudate, follicle, lid swelling, and chemosis were mostly found on examination.

## DISCUSSION

The prevalence of acute conjunctivitis in our study was 1, 240 (22.52%) which is higher compared to a similar previous study where the prevalence was 8.1%.^6^ Similarly, the prevalence of conjunctivitis in our study is higher than a study previously conducted in Kolkata with a prevalence of 17.23%.^7^ The prevalence might have been higher due to the epidemic of conjunctivitis at the time of the study. This descriptive study had a representation of patients of all age groups and both sexes.

Conjunctivitis is also referred to as pink eye,^8^ which concurs with the findings of our study. Redness, eye swelling, watering, discomfort, and discharge were symptoms with a higher frequency in this study, which is also supported by the findings of a systematic review.^9^ Conjunctivitis can be caused by various factors, leading to distinct types of conditions, each with unique causes, clinical features, and treatment approaches.^10^

The first manifestation of a viral infection may be conjunctivitis, which can also be the only manifestation.^11^ A papillary conjunctival reaction, or pseudomembranous conjunctivitis, strongly suggests a bacterial origin for conjunctivitis, while a follicular conjunctival reaction is more likely to indicate a viral cause. Viral conjunctivitis is the most common overall cause of infectious conjunctivitis, which often starts suddenly and can be highly contagious, resulting in outbreaks, especially in crowded settings.^8,12^

Enterovirus multiplies in the intestines and spreads through respiratory secretions, faeces, and contaminated water. A study has also reported an association between the quality of the water supply and enterovirus infection.^13,14^ With this in mind, the role of the water supply in Kathmandu Valley in the outbreak may be the subject of further study. Overcrowding, poorer ventilation of the accommodation, and lesser body immunity might be other parameters that had affected the disease and the outcome.

After viral aetiology, bacterial conjunctivitis is commonly encountered. The most common pathogens for bacterial conjunctivitis in adults are staphylococcal species.^15^ Bacterial conjunctivitis has a course of 1-2 weeks, and its presentation includes features like redness of the eye, chemosis, and mucopurulent discharge. Bacterial conjunctivitis can be differentiated from viral conjunctivitis through the clinical features, but one can always benefit from the testing kits.^8^ It is necessary to differentiate bacterial from viral causes to avoid unnecessary antibiotic use.

The medical intervention aims primarily at controlling the large outbreaks as well as instituting preventative measures to protect vulnerable groups, such as children, the elderly, pregnant women, and immunocompromised individuals, by encouraging frequent hand-washing and reducing contact with the affected individuals. Restriction of touching the eyes with infected hands helps to prevent and reduce the flaring up of the disease.^12^

This is a single-centre study with a limited sample size and duration. Moreover, the limitation in quota regarding the opportunity of virus detection facilities with PCR testing and the availability of PCR kits in low- and middle-income countries like Nepal was a big limitation in generalizing the findings of the study.

## CONCLUSIONS

The prevalence of acute conjunctivitis among patients visiting the Department of Opthalmology was found to be higher than other studies done in similar settings.
